# Quantum Fluctuations of a Superconductor Order Parameter

**DOI:** 10.1186/s11671-016-1582-7

**Published:** 2016-08-17

**Authors:** K. Yu Arutyunov, J. S. Lehtinen

**Affiliations:** 1 National Research University Higher School of Economics, Moscow Institute of Electronics and Mathematics 101000, Moscow, Russia; 2 VTT Technical Research Centre of Finland Ltd., Centre for Metrology MIKES, P.O. Box 1000, Espoo, FI-02044 VTT Finland

**Keywords:** Quasi-one-dimensional superconductivity, Quantum fluctuations, Tunneling

## Abstract

Tunneling I–V characteristics between very narrow titanium nanowires and “massive” superconducting aluminum were measured. The clear trend was observed: the thinner the titanium electrode, the broader the singularity at eV = Δ_1_(Al) + Δ_2_(Ti). The phenomenon can be explained by broadening of the gap edge of the quasi-one-dimensional titanium channels due to quantum fluctuations of the order parameter modulus |Δ_2_|. The range of the nanowire diameters, where the effect is pronounced, correlates with dimensions where the phase fluctuations of the complex superconducting order parameter Δ = |Δ|e^iφ^, the quantum phase slips, broadening the R(T) dependencies, have been observed.

## Background

Over several decades, the tendency for miniaturization of electronic devices could have been described by the Moore’s law: the number of elements on microchips doubles each 18 months. However, nowadays, all authorities (including Gordon Moore himself, the founder of *Intel*) agree that the Moore’s law soon will come to saturation. According to various prognoses, further integration of commercial nanoelectronic elements is expected to reach stagnation by 2016–2018. Basically, one can figure out two main reasons for such pessimistic forecast. The first one is purely technologic: the dramatic increase of heat dissipation per unit volume (or area). The second reason comes from fundamental properties of electron transport in solids: below certain scales, the behavior of ultra-small elements (rough estimation is about 10 nm) becomes qualitatively different from the properties of macroscopic conductors. In this limit, various quantum phenomena take place driving the device out of conventional (classic) operation mode.

One can naively suggest that a radical solution of the first problem might be the utilization of superconducting elements dissipating zero heat. Indeed already now, superconducting nanoelectronic devices are widely used in various high-tech applications: ultra-sensitive detectors of electromagnetic radiation (e.g., bolometers or transition edge sensors), electric voltage standards utilizing Josephson effect, and quantum bits. Hence, one may tend to use nanoscale superconductors as building blocks of the new generation of nanoelectronic devices. However, already now, there exist both experimental and theoretical studies [1] claiming that below certain scales (of the order of 10 nm), the properties of nanoscale superconductors qualitatively differ from the properties of macroscopic objects. The main reason is the impact of fluctuations which become more pronounced with reduction of the system dimension(s). In addition to undesired contribution “killing” the dissipationless superconducting state, quantum fluctuations are expected to give rise to qualitatively new effects and, correspondingly, should lead to the new generation of nanoelectronic devices.

It is well-known that superconductivity can be described in terms of complex order parameter Δ = |Δ|e^iφ^. Following the quantum nature of superconductivity, the order parameter can exhibit both classic and quantum fluctuations. The classic (thermal) contribution is important sufficiently close to critical temperature *T*_c_, while the impact of quantum fluctuations should be non-negligible within the whole temperature range, including the low-*T* limit *T* << *T*_c_.

It has been realized that quantum fluctuations of the order parameter Δ = |Δ|e^iφ^ can dramatically modify the properties of sufficiently narrow superconducting channels [1]. The specific manifestation of the phenomenon, related to fluctuations of the phase φ, is called *quantum phase slip* (QPS). The process corresponds to momentary zeroing of the modulus |Δ| and simultaneous “slip” of the phase φ by ±2π. This leads to several non-trivial effects: finite resistance at temperatures well below the critical point [2–4], suppression of persistent currents in narrow nanorings [5], and coherent superposition of QPSs [6–11]. Here, we present our studies of the related phenomenon-quantum fluctuations of the modulus |Δ| of the order parameter in thin titanium nanowires. We show that the range of the nanowire diameters, where the effect is pronounced, correlates with dimensions where the QPSs, broadening the *R*(*T*) dependencies, have been observed.

### Theory background

The subject of QPSs has been discussed rather extensively [1, 12, 13]. The rate of QPSs can be expressed as:

1 $$ {\varGamma}_{\mathrm{Q}\mathrm{PS}}=\frac{\left|\varDelta \right|}{h}\frac{R_{\mathrm{Q}}}{R_{\mathrm{N}}}{\left(\frac{L}{\xi}\right)}^2 \exp \left(-{S}_{\mathrm{Q}\mathrm{PS}}\right) $$

where ξ is the coherence length and *L* is the nanowire length. The QPS action is *S*_QPS_ = *A*[*R*_Q_/*R*_N_][*L*/*ξ*(*T*)], where *R*_N_ is the sample resistance in normal state, *R*_Q_ = *h/*(2*e*)^2^ = 6.45 kΩ is the superconducting quantum resistance, and the constant *A* ≈ 1 is the numerical factor that unfortunately cannot be determined more precisely within the model [12, 13]. The impact of fluctuations exponentially strongly depends on the cross section of a superconductor channel. It can be easily shown that for a given (small) cross section of a nanowire, materials with low critical temperature and high normal state resistivity are of advantage for observation of the QPS effect [1]. In particular, it has been shown that superconducting titanium is the material where QPS effects do exist [4, 5, 7, 9–11].

The magnitude of the related effect—fluctuations of the modulus |Δ|—is determined by the same QPS action [1]:

2 $$ \frac{\delta \left|\varDelta \right|}{\left|\tilde{\varDelta}\right|}=\frac{1}{S_{\mathrm{QPS}}} $$

where $\left|\tilde{\varDelta}\right|$ stands for the mean value of the order parameter modulus. For ultra-thin superconducting nanowires, where the QPS contribution has been observed, the corresponding effect should be measurable. For example, in titanium nanostructures [4, 5, 7, 9–11], the magnitude of the order parameter modulus might reach an impressive value of ~20 %.

## Methods

As the modulus of the order parameter corresponds to the energy gap in excitation spectrum of a superconductor, a straightforward experimental approach would be to measure RF absorption (or reflection) spectra of a quasi-1D nanowire (or an array of similar nanowires) of relevant cross section. The task is doable, but it requires an appropriate expertise and complicated RF equipment that the authors do not have at their disposal. Hence, an alternative approach was selected. It is a common knowledge that I–V tunnel characteristics of a superconductor-insulator-superconductor (SIS) or normal metal-insulator-superconductor (NIS) junction has a singularity at energies eV corresponding to the energy gap of the superconductor(s) [14]. Of particular interest is the S_1_IS_2_ configuration, as it provides “sharp” singularity at eV = Δ_1_(T) + Δ_2_(T), which is almost temperature independent in the low-*T* limit *T* << *T*_c1,2_. In practice, the I–V dependencies do demonstrate a certain broadening of the Δ_1_(T) + Δ_2_(T) singularity. In addition to the experimental artifacts, undesired impact of EM environment, junction inhomogeneity, etc., the non-zero width of the singularity can be attributed to finite quasiparticle lifetimes, characterized by the *Dynes parameter* Γ [15]. However, in absolute terms, the mentioned contribution can be made rather small. For example, in aluminum-based SIS structures [16], one can obtain experimental broadening $\delta \left|{\varDelta}_1+{\varDelta}_2\right|/\left|{\tilde{\varDelta}}_1+{\tilde{\varDelta}}_2\right| < {10}^{-3}$, which is much smaller than the magnitude of the effect we are searching for.

**Fig. 1:**
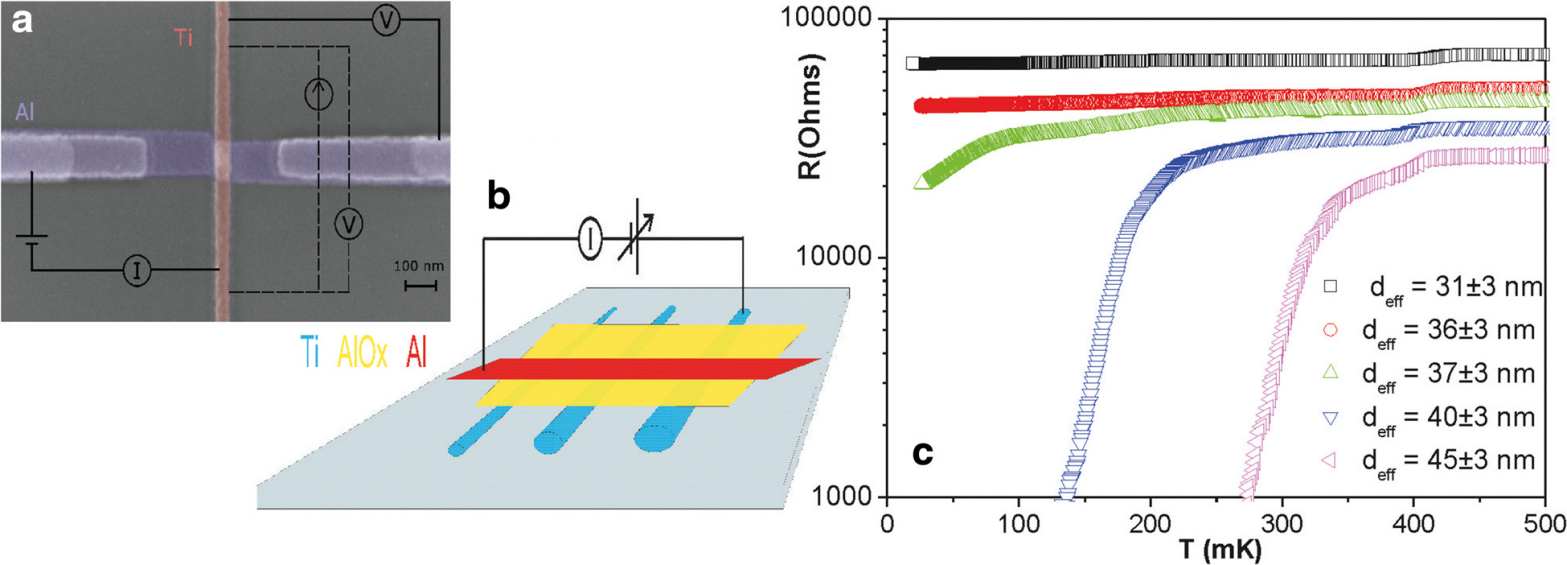
**a** SEM image of a typical *Al-AlO*_*x*_ *-Ti* tunnel junction with schematic of the electric circuit. **b** Schematic of the nanostructure layout. **c** R(T) dependencies of titanium nanowires with various effective diameters *d*_eff_

To proceed, we fabricated S_1_IS_2_ nanostructures, where S_1_ stands for the “massive” aluminum electrode and S_2_ corresponds to counter electrodes in a shape of thin titanium nanowires of various diameters (Fig. 1a, b). The samples were fabricated using PMMA/MAA double-layer lift-off e-beam lithography and directional ultra-high vacuum metal deposition. The nanostructures were formed on the surface of the oxidized silicon. The tunnel barrier “I” was formed by oxidation of the aluminum layer prior to deposition of titanium. Scanning electron and atomic force microscope analyses were used to test the samples. Only those structures which contained no obvious artifacts were further processed. Electron transport measurements were made at ultra-low temperatures in ^3^He^4^He dilution refrigerator located inside EM-shielded room. All input/output lines contained multi-stage RLC filters to reduce the impact of noisy EM environment [17].

## Results and Discussion

In addition to V–I dependencies, the R(T) characteristics of each titanium nanowire were measured. In accordance with earlier experiments [4, 5, 7, 9–11]; relatively thick samples demonstrated sharp phase transitions, while the thinnest nanowires with an effective diameter < 40 nm, the R(T) transitions were broadened, and their shape could be attributed to the QPS effect (Fig. 1c). The observation supports the expectation that quantum fluctuations are present in the thinnest titanium nanowires studied in this work.

**Fig. 2:**
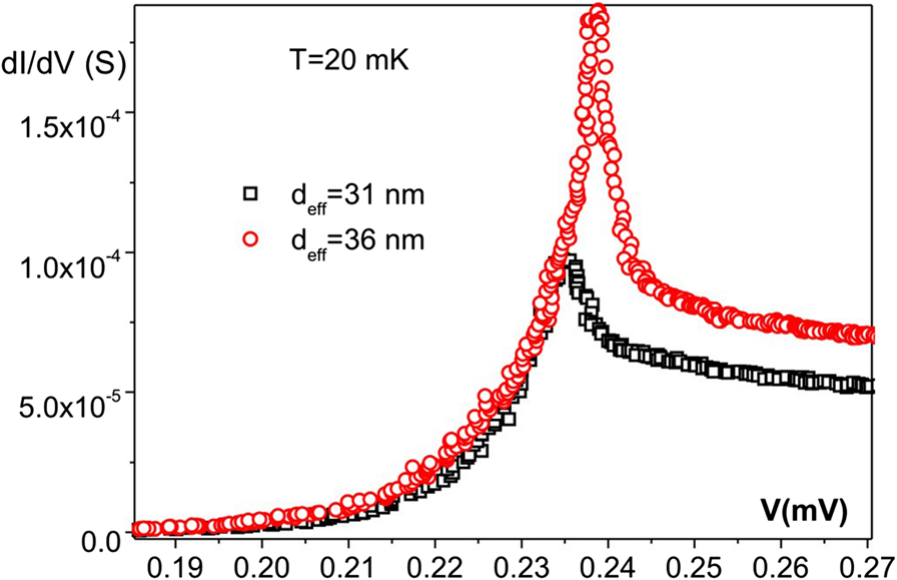
First derivative dV/dI characteristic: blow-up of the singularity at eV = Δ_1_(Al) + Δ_2_(Ti) of two S_1_IS_2_ junctions with two titanium electrodes of different effective diameters *d*_eff_ equal to 31 and 36 nm (indicated in the *inset*). One can clearly see that for the thinnest titanium electrode, the absolute value of the mean gap $\left|{\tilde{\varDelta}}_2\right|$ is smaller and the broadening $\delta \left|{\varDelta}_2\right|/\left|{\tilde{\varDelta}}_2\right|$ is larger

The I–V dependencies between the aluminum S_1_ and each of the titanium nanowires S_2_ (Fig. 1b) were the primary subjects of the study. The I–V characteristics were measured at temperatures above and below the critical temperature of thin film superconducting titanium *T*_c_(Ti) ≈ 400 mK. Above the critical temperature of titanium, but below the critical temperature of aliminum *T*_c_(Al) ≈ 1.4 K, no unexpected features were detected: just a conventional I–Vs of a S_1_IN_2_ junction. At low temperatures *T* << *T*_c_(Ti), the I–Vs demonstrated the overall expected shape for a S_1_IS_2_ junction (Fig. 2). However, the clear trend was observed: the thinner the S_2_ (titanium) electrode, the broader the singularity at eV = Δ_1_(Al) + Δ_2_(Ti).

**Fig. 3:**
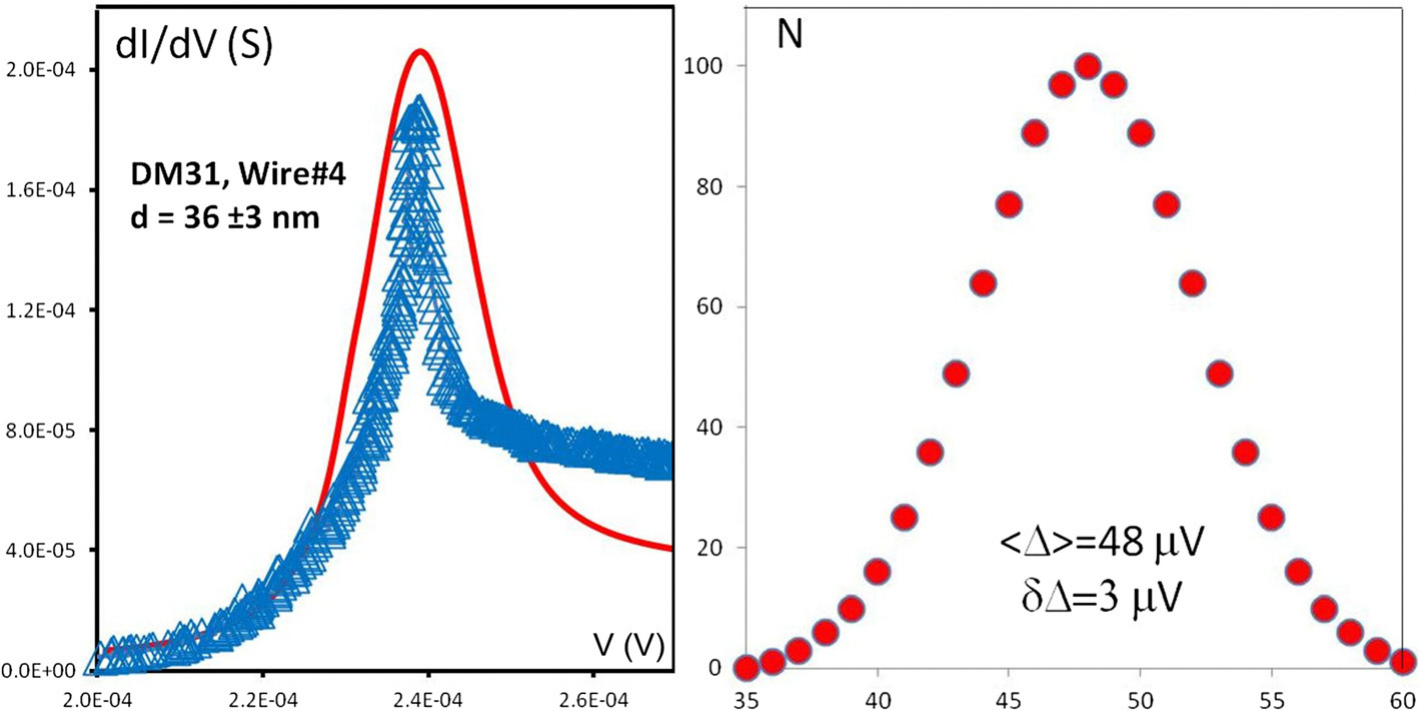
**a** Experimental (*symbols*) and simulated (*line*) for a typical gap-edge singularity eV = Δ_1_(Al) + Δ_2_(Ti). **b** Distribution of the titanium gap fluctuations used in fitting data from panel **a**: $\left|{\tilde{\varDelta}}_2\right|=48\;\upmu \mathrm{e}\mathrm{V}$, *δ*|*Δ*_2_| = 3 μeV

To account for the observation, we have simulated the I–V dependencies using conventional expression for the tunnel current [14] of a voltage-biased S_1_IS_2_ junction (Fig. 3a) with finite “smearing” of the gap Δ_2_(Ti) assuming Gaussian distribution of the fluctuations (Fig. 3b). Certainly, the utilized model is essentially phenomenologic and does not take into consideration other possible reasons for smearing of the singularity at eV = Δ_1_(Al) + Δ_2_(Ti) [16]. However, as all titanium electrodes were fabricated in one experimental run and the measurements were performed within single cool down, one can reasonably assume that “intrinsic” reasons for the singularity broadening (e.g., finite Dynes parameter) should be the same. Hence, one can conclude that what is observed is somehow related to a size effect. We believe that the observation can be explained by the size-dependent contribution of quantum fluctuations of the order parameter modulus |*Δ*_2_|. We hope that our experiments will stimulate further theory studies. Presumably, a comprehensive microscopic theory, in addition to smearing of the gap edge, should also self-consistently calculate the renormalization of the density of states and the distribution function of a superconductor due to quantum fluctuations of the order parameter.

## Conclusions

Tunneling I–V characteristics S_1_IS_2_ between “massive” aluminum electrode S_1_ and several titanium nanowires S_2_ were measured. For the thinnest titanium samples, the clear trend was observed: the thinner the S_2_ (titanium) electrode, the broader the singularity at eV = Δ_1_(Al) + Δ_2_(Ti). We attribute the observation to contribution of quantum fluctuations of the order parameter modulus |*Δ*_2_| of the thin titanium nanowires. The range of the nanowire diameters where the effect is observed correlates with dimensions where the contribution of the phase fluctuations—the quantum phase slips, broadening the R(T) dependencies, have been observed.
